# Complicated Traumatic Pneumothorax Requiring VATS With Lobectomy and Pleurodesis Arising Following SARS-CoV-2 (COVID-19) Recovery

**DOI:** 10.7759/cureus.49238

**Published:** 2023-11-22

**Authors:** Auda H Auda, Marco Lawandy, Omead J Mirgoli, Mohammad Jurri, Iyad Baker, Elizabeth August, Tayyab Malik, Faryaal Bokhari, Ayrton I Bangolo

**Affiliations:** 1 Family Medicine, Hackensack Meridian Health Palisades Medical Center, North Bergen, USA; 2 Internal Medicine, Hackensack Meridian Health Palisades Medical Center, North Bergen, USA

**Keywords:** video-assisted thoracoscopic surgery (vats), pleurodesis, pulmonary lobectomy, pneumothorax (ptx), covid-19

## Abstract

A case is presented in which COVID-19 pneumonia led a young male patient to develop a pneumothorax requiring lobectomy and pleurodesis after the resolution of COVID-19 pneumonia. The literature review showed a few similar cases with clear evidence suggesting that prior COVID-19 infection may be considered a risk factor for pneumothorax. It is crucial for clinicians to take such risk factors into consideration for better clinical outcomes.

## Introduction

Pneumothorax is a collection of air outside the lung but within the pleural cavity that can apply pressure toward the lung causing it to collapse. The result can be secondary to chest wall injury, underlying pulmonary disorder, or active infection such as COVID-19. While the patient’s history and physical exam can often lead us to an underlying cause, there have been many cases of reported spontaneous pneumothoraces after contraction of COVID-19 with several cases presenting after the disease resolution [1]. Here, we describe the case of a 27-year-old man. He had a mild COVID-19 infection 11 months prior to presentation and was admitted to the hospital due to right-sided chest pain and worsening shortness of breath caused by a traumatic pneumothorax. Following chest tube placement with subsequent lobectomy and pleurodesis, the patient’s pneumothorax resolved. This case report aimed to encourage clinicians to include traumatic pneumothorax in their differential diagnosis in any patient with worsening or new-onset respiratory distress or chest pain, even after the resolution of COVID-19.

## Case presentation

A 27-year-old male with a history of mild COVID-19 infection 11 months prior to presentation was admitted to the hospital for right-sided chest pain, worsening shortness of breath, and generalized body aches. 

The patient was in his usual state of health until one day prior to admission. He was involved in a physical altercation with a family member in which he fell to the ground onto the right side of his body, and the other individual fell on top of him. Subsequently, the patient experienced excruciating right-sided chest pain, shortness of breath, and generalized body aches, which worsened over time.

On initial presentation, the patient’s vital signs were stable with a weight of 188 lbs, height of 5 ft 10 in, BMI of 27.0 kg/m², temperature of 36.6°C, blood pressure of 129/76, pulse of 74 and regular, respiratory rate of 20, and oxygen saturation of 97% breathing ambient air. Right flank ecchymosis was noted. Pulmonary effort and breath sounds were normal; there was no crepitus or wheezing. However, there was right chest wall tenderness and pain with deep inspiration.

**Figure 1:**
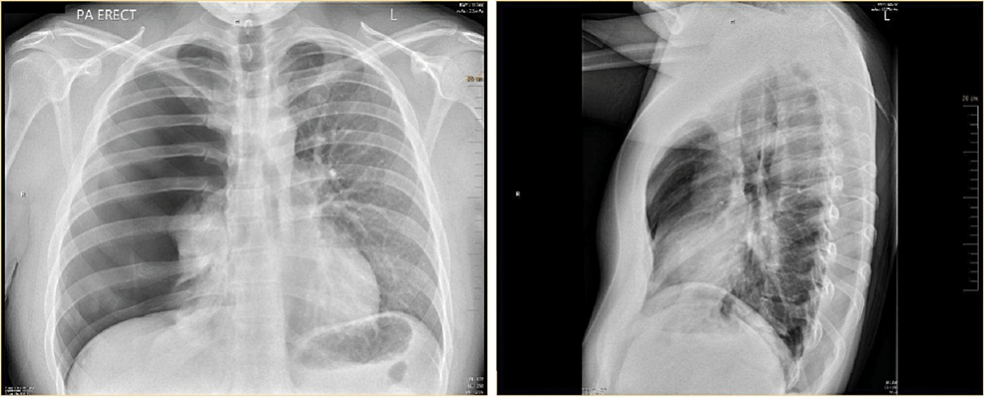
Posteroanterior and lateral chest radiographs upon hospital admission showing large right pneumothorax

**Figure 2:**
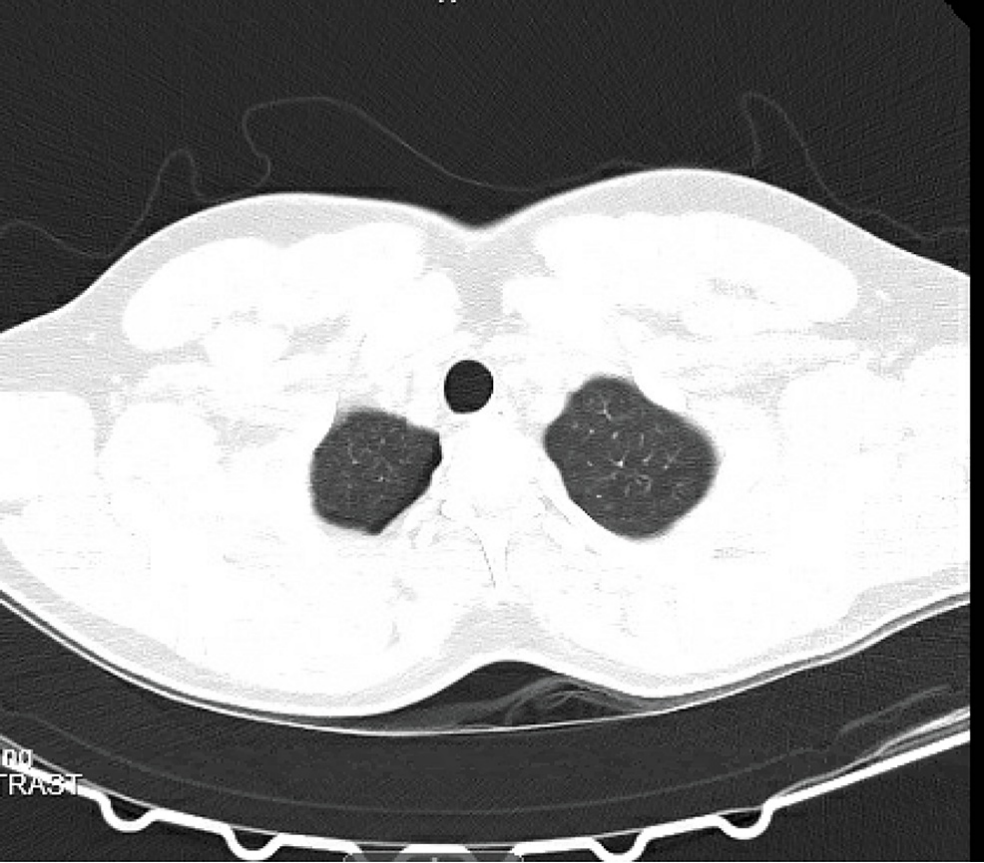
Computed tomography showing small remaining right apical pneumothorax

**Figure 3:**
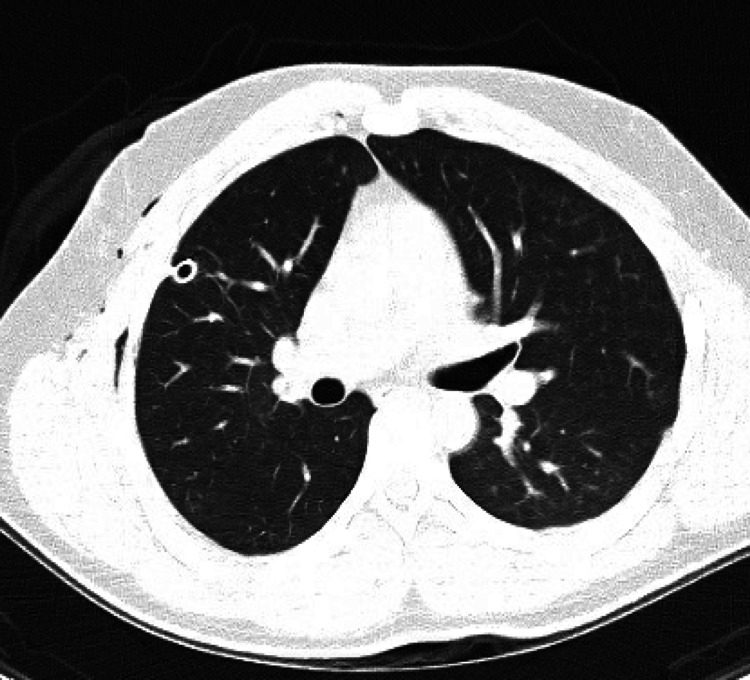
Computed tomography showing subcutaneous emphysema in the right chest wall

Posteroanterior and lateral chest radiographs revealed a large right pneumothorax and complete atelectasis of the right lung with no acute displaced rib fracture and normal cardiac and mediastinal contours (Figure 1). The patient’s condition was deemed emergent, and a right-sided chest tube was placed. One hour later a portable chest X-ray (CXR) showed a small right pneumothorax, diminished compared to previous imaging, with improved aeration of the right lung field. The right chest tube was in a stable position. A subsequent chest computed tomography confirmed the presence of a small residual right apical pneumothorax along with subcutaneous emphysema in the right chest wall. There were no signs of pleural or pericardial effusion, and the heart size and mediastinal structures appeared normal, without any bullae or blebs (Figure 2 and Figure 3).

The patient was admitted to the hospital for continued treatment and observation, and his condition generally improved. On day 3, the chest tube was found to be draining serosanguinous fluid with a noted tiny air leak. A repeat chest radiograph revealed a new moderate right-sided pneumothorax and right basilar atelectasis. The chest tube was connected back to suction. A repeat CXR later showed right basilar atelectasis and a trace right basilar pneumothorax that had significantly decreased in size. 

**Figure 4:**
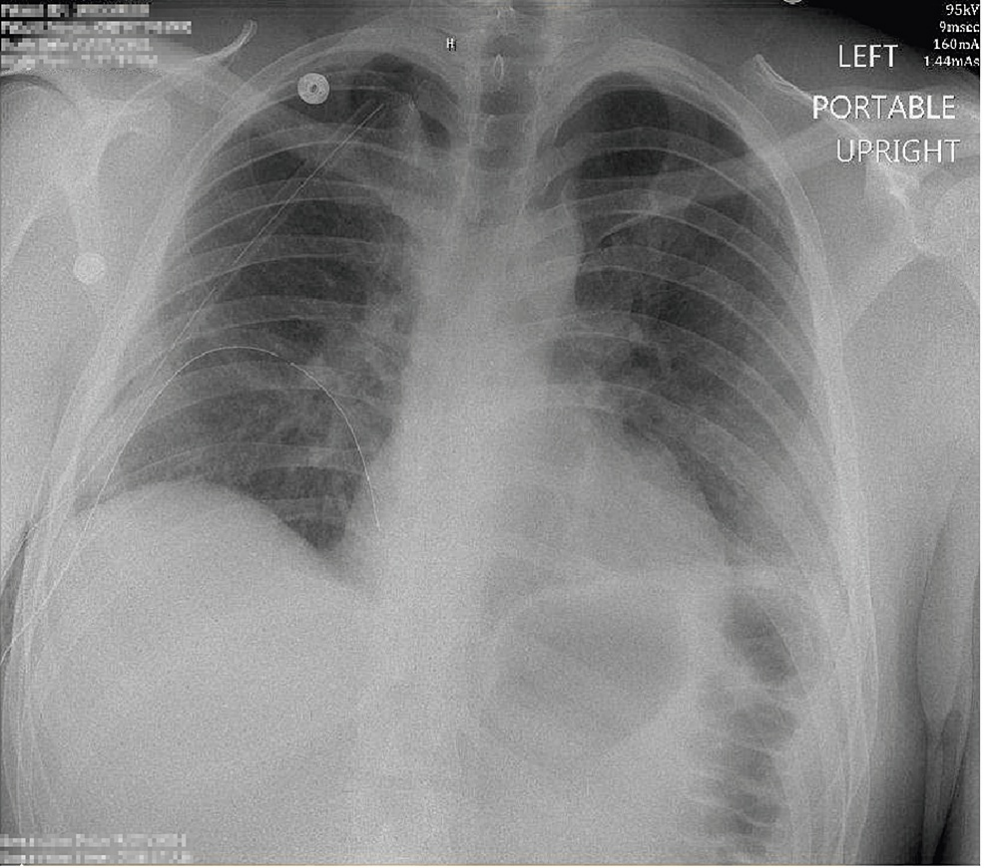
Portable CXR on day 6 demonstrating interval partial right upper lobe resection. Parenchymal opacity in the medial right upper lung is likely postsurgical in nature. There is a small right apical pneumothorax. Right apical and basilar chest tubes are present. The left lung demonstrates ill-defined perihilar and basilar opacities, which may represent atelectasis or asymmetric edema. Cardiomediastinal contours are unremarkable

**Figure 5:**
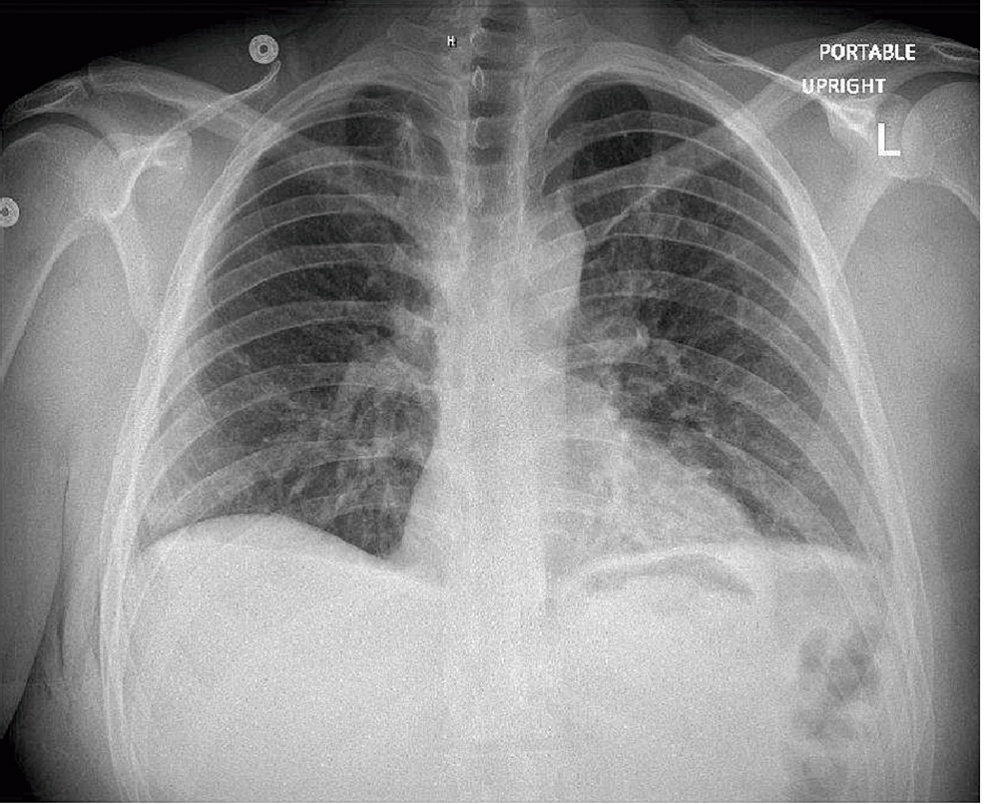
CXR on the day of discharge showing resolution of pneumothorax CXR, chest X-ray

**Figure 6:**
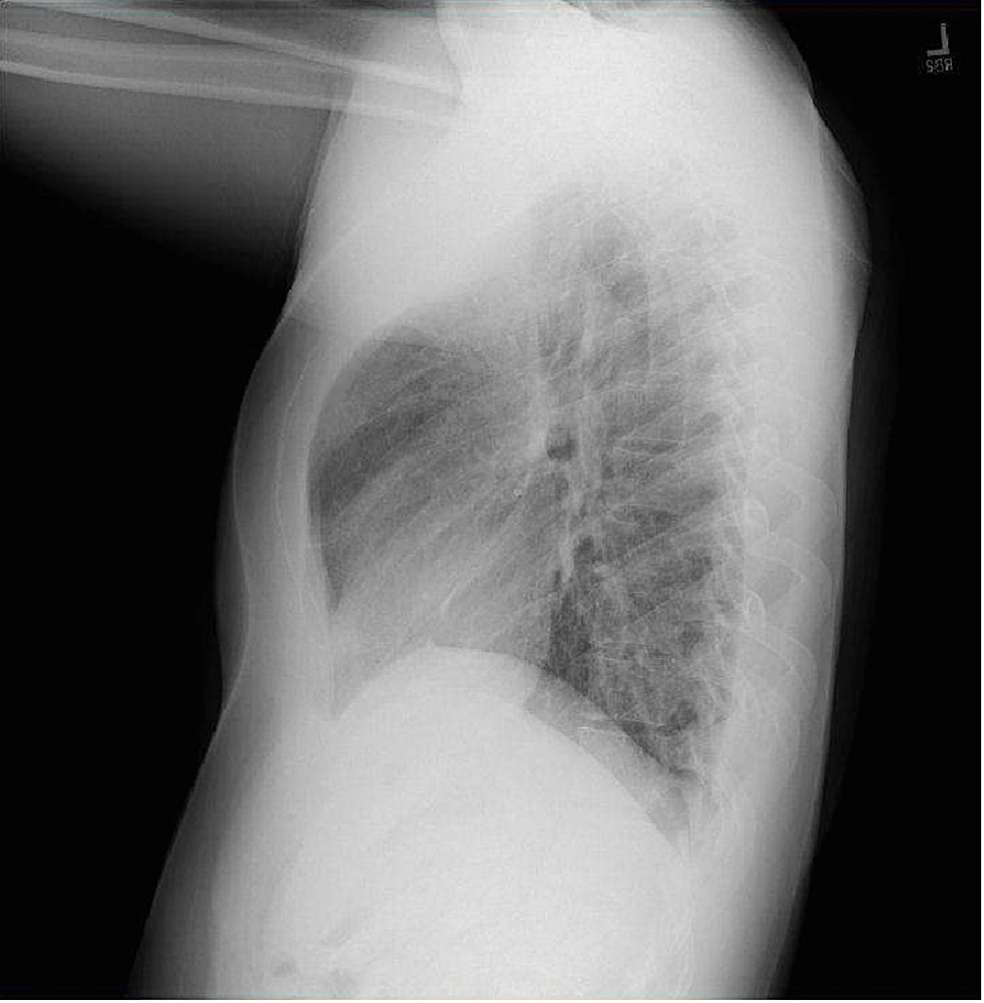
Follow-up anteroposterior and lateral radiographs at two-week follow-up visit demonstrating sustained resolution of the pneumothorax

On day 5, a right video-assisted thoracoscopic surgery (VATS) with right upper lobe lobectomy was performed as well as mechanical and chemical pleurodesis with doxycycline. Sequential CXRs over the following three days showed two right thoracostomy tubes in the proper position and a clear right chest (Figure 4 and Figure 5). The patient’s pain resolved and on day 8, he was determined to be medically optimized for discharge with outpatient management. A radiograph taken at a follow-up visit two weeks later confirmed the resolution of the pneumothorax (Figure 6) and the patient remained asymptomatic.

## Discussion

This is a case of a 27-year-old male with a history of COVID-19 infection who presented to the emergency department with excruciating right-sided chest pain and shortness of breath after a traumatic injury to his chest wall. The patient was subsequently diagnosed with right-sided pneumothorax and treated with a right-sided chest tube and elective VATS with right upper lobectomy and pleurodesis.

After initial stabilization and treatment of pneumothorax, multidisciplinary decisions are to be made whether the patient is at increased risk of pneumothorax recurrence. If so, definitive treatment is required to prevent such recurrence [2-4]. Most commonly a surgical approach is recommended using VATS to perform mechanical or chemical pleurodesis [2-4]. Pleurodesis is a thoracic surgical procedure performed using either mechanical or chemical irritants to produce pleural inflammation and fibrosis that adheres the pleural layers together, thus preventing future pneumothorax. The choice of procedure should be individualized to each patient and also depends on the available resources and technical expertise. Although open thoracotomy in addition to pleurectomy has shown the lowest recurrent rate, VATS is still preferred in most cases, because it is more tolerable and associated with less recovery time and postoperative complications [2-4].

Development of a pneumothorax can occur spontaneously at any time during the course of an underlying pulmonary disorder, injury, or during an active infection, such as COVID-19. Risk factors for the development of a spontaneous pneumothorax include male gender, chronic obstructive pulmonary disease, cystic fibrosis, tall stature, cigarette/marijuana smoking, malignancy, structural abnormalities such as Marfan syndrome or Ehler-Danlos syndrome, or pulmonary infections [1].

While there are many cases of spontaneous pneumothorax development shortly after contraction of a symptomatic COVID-19 infection, they are much less frequently reported after disease resolution. A brief literature search yielded only 25 cases, one of which was a 37-year-old man with no past medical history hospitalized for pneumonia secondary to COVID-19 and acute hypoxemic respiratory failure requiring a high-flow nasal cannula. The patient developed a right-sided pneumothorax requiring chest tube placement two weeks after disease onset and two days after hospital discharge [5]. Among the 25 cases, the average time of development of pneumothorax from the onset of the COVID-19 symptoms was 19 days, which implies that most of the cases of pneumothorax were later in the disease course or even after the resolution of the initial COVID-19 [1]. This suggests that a pneumothorax should be on the differential in a patient with worsening or new-onset respiratory distress or chest pain, even after the resolution of COVID-19.

## Conclusions

Elucidated is a case in which a juvenile male patient manifested pneumothorax necessitating lobectomy and pleurodesis approximately 11 months subsequent to the resolution of COVID-19 pneumonia. Upon conducting a concise examination of the extant literature, a paucity of comparable cases was observed, accompanied by compelling evidence intimating that antecedent infection with COVID-19 may be regarded as a predisposing factor for the development of pneumothorax.
